# Analysis of the kinetics of the parathyroid hormone, and of associated patient outcomes, in a cohort of haemodialysis patients

**DOI:** 10.1186/s12882-016-0365-9

**Published:** 2016-10-18

**Authors:** Guillaume Jean, Jean-Claude Souberbielle, Eric Zaoui, Christie Lorriaux, Jean-Marc Hurot, Brice Mayor, Patrik Deleaval, Manolie Mehdi, Charles Chazot

**Affiliations:** 1 NEPHROCARE Tassin-Charcot, 7, Avenue Maréchal FOCH, 69110 Sainte Foy-Les-Lyon, France; 2 Université Paris Descartes, Inserm U845, and Hôpital Necker, Service d’explorations fonctionnelles, Paris, France; 3 NOVESCIA Rhône-Alpes, Laboratoire du Grand Vallon, 69110 Sainte Foy-les-Lyon, France

**Keywords:** Parathyroid hormone, Survival rate, Haemodialysis, Parathyroidectomy, Calcium, Vitamin D, Calcimimetics

## Abstract

**Background:**

Observational studies have recently associated a decrease in serum parathyroid hormone (PTH) level with a higher rate of mortality among hemodialysis (HD) patients. Decreases in PTH level can result from medical intervention (MPD) and surgical parathyroidectomy (PTX), or may occur spontaneously, usually associated with an underlying malnutrition-inflammation syndrome (SPD). The aim of our study was to prospectively identify the incidence of decreases in PTH level in a cohort of HD patients and the frequency distribution of the different causes (MPD, PTX and SPD), as well as to evaluate the survival outcomes for each PTH group (MPD, PTX and SPD) compared to patients who did not experience a PTH decrease over the first 36 months of the study (NPD).

**Methods:**

The 197 patients receiving HD at our center in January 2010, and meeting our eligibility criteria, were enrolled in our prospective study, and were observed for a period of 60 months. A decrease in PTH level >50 % between two successive PTH measurements obtained within an interval <3 months was defined as a significant event. MPD referred to a decrease in PTH due to an increased oral calcium intake, increased dialysate calcium concentration (DCC), increased alfacalcidol use, or use of cinacalcet therapy. A surgical 7/8 PTX was performed in young patients or in patients in whom cinacalcet therapy failed. SPD referred to a decrease in PTH related to a medical or surgical event. Baseline characteristics among patients in each group (MPD, PTX, SPD, and NPD) were evaluated using Fisher’s exact test. The 60-month survival was evaluated using Kaplan-Meier and Cox multivariable proportional hazards models. Univariate and multivariate Cox analyzes were used identify variables with mortality. The relative risk of mortality was expressed as a hazard ratio (HR).

**Results:**

The distribution of the 197 patients forming our four study groups was 34 % in the NPD group, 35 % in the SPD group, 25 % in the MSD group and 6 % in the PTX group. Among patients in the SPD group, the main acute comorbid conditions were peripheral vascular and cardiac complications, sepsis, fractures, and cancers with an increase in serum CRP level (from 14.3 ± 18 to 132 ± 90 mg/L) and a decrease in serum albumin (from 33 ± 4.5 to 28.6 ± 4 g/L). In the MPD group, the main therapeutic change was an increase in DCC, either independently or in association with cinacalcet therapy. The median survival rate among patients was 10 months for SPD, compared to 22 months among patients in the MPD group (*p* < 0.001). Using multivariable Cox model and taking the NPD group as reference, the risk of mortality was lower among patients in the MPD group (HR, 0.42[0.2-0.87] *p* = 0.01), with survival being comparable for the SPD and NPD groups (HR, 1.3 [0.75-2.2]). No mortality was observed in the PTX group.

**Conclusion:**

The poor outcomes associated with SPD, related to acute comorbid conditions, should not lead to undertreat secondary hyperparathyroidism whose appropriate medical or surgical therapies are associated with better outcomes.

## Background

Secondary hyperparathyroidism (SHPT) is a common comorbidity in patients with chronic kidney disease (CKD) and, particularly in those on hemodialysis (HD) [1]. Due to an unpredictable bone resistance to parathyroid hormone (PTH) action, maintaining a mild biological SHPT is recommended in most HD patients to favor a normal bone turnover rate. According to the most recent international Kidney Disease Improving Global Outcomes guidelines, experts recommend target serum PTH levels of 2 to 9 times the upper normal limit of the assay in HD patients [2]. Identification of bone turnover rate from serum PTH levels, however, is not straightforward. Bone histology studies have shown that SHPT can be excluded by lower serum PTH levels and adynamic bone disease (ABD) by higher serum PTH levels [3]. Between these two limits exists a ‘grey zone’ in which normal bone, ABD and SHPT can all be observed. Therefore, the assessment of serum bone markers and even bone biopsies, at times, are necessary to better assess the rate of bone turnover in HD patients.

Besides, the KDIGO experts suggest that “marked changes in PTH levels in either direction within the target range prompt an initiation or change in therapy to avoid progression to levels outside of this range (2C)”[2].

The Dialysis Outcomes and Practice Patterns Study (DOPPS) provided evidence of an increase in mean serum PTH level being an important health issue in HD patients [4].

Beyond having consequences on the rate of bone turnover, elevated PTH is also a well-known uremic toxin which has been associated with cardiovascular calcification [5] and mortality [4]. However, it is important to note that both low [6] and high [7] serum PTH levels have been associated with poor health outcomes in HD patients. While low PTH levels commonly result from surgical parathyroidectomy (PTX) and the prescription of calcimimetics, various other causes can result in low PTH levels, including an excessive calcium and vitamin D load and hypercalcemia [8, 9], as well as the malnutrition-inflammation syndrome [10–13] and prolonged immobilization [14].

Recently, two observational studies have reported an increased rate of mortality associated with low PTH levels [15, 16]. However, as these two studies failed to identify the underlying cause of low PTH levels, we believe that their suggestion of a possible risk in treating SHPT in HD patients might be misleading. To address this issue, we undertook our study to prospectively identify the incidence of PTH decrease in a cohort of HD patients and to describe the frequency distribution of the different causes of decreased PTH in this clinical cohort. A second aim of our study was to evaluate survival outcomes associated with different identified causes of decreased PTH and in patients with no PTH decrease throughout their course of treatment.

## Methods

All patients on HD at our institution in January 2010 were screened for eligibility using the following exclusion criteria: dialysis vintage <6 months and previous surgical parathyroidectomy (PTX). Patients meeting our eligibility criteria were enrolled into the study and observed for a period of 60 months. The study was conducted in compliance with the Declaration of Helsinki, and all patients provided their signed consent to have their data entered in a database for analysis.

**Fig. 1:**
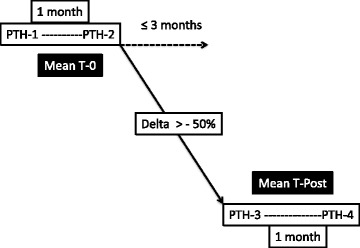
Protocol design

The pre-dialysis, mid-week, serum PTH level was sampled every month. For analysis, patients were classified by the presence or absence of an event of significant decrease in serum PTH level over the first 36 months of the study. A significant decrease in serum PTH level was defined as a decrease of more than 50 % between two measurement periods within an interval of less than 3 months, with the mean of two successive PTH values used to calculate the change in PTH (Fig. 1). This was based on the study from Cavalier et al. reporting that at least 40 % difference between PTH measurements is required from their intraindividual variability study [17].

A *surgical PTH decrease* was defined as a decrease in serum PTH level identified after surgical subtotal (7/8) parathyroidectomy (PTX). A *medical PTH decrease* (MPD) was defined as a decrease in serum PTH level associated with the use of one or more treatments known to decrease PTH, including: native vitamin D supplementation; prescription of calcitriol, or of its analogs, oral calcium, and cinacalcet; and increased dialysate calcium concentration (DCC). A *spontaneous PTH decrease* (SPD) was defined as a decrease in serum PTH level without any change in therapy. The underlying clinical condition was recorded together with serum levels of albumin and C-reactive protein (CRP). Patients with no identified decrease in PTH were classified in the *no PTH decrease* (NPD) group.

Patients were dialyzed thrice-weekly, 4 to 8-h per session, using polysulfone high-flux filters (FX 60, 80, 100, 800 and 1000; Fresenius Medical Care©, Bad Homburg, Germany) in HD or post-dilution online hemodiafiltration (HDF). Blood flow rate ranged from 220 to 400 ml/min, and the dialysate flow rate ranged from 350 to 800 ml/min. The standard dialysis calcium concentration was 1.5 mmol/L. However, a 1.25 mmol/L concentration was prescribed for patients with low PTH levels (<100 pg/mL), and a 1.75 mmol/L concentration was recommended in cases where the PTH levels were high (>400 pg/mL). Our strategy to optimize mineral and bone status during HD has been previously reported [18], and has remained stable for years.

The rate of bone turnover was assessed using the bone markers: bone alkaline phosphatase (b-ALP) and Beta(ß)-crossLaps (CTX). The prevention and treatment of SHPT included systematic cholecalciferol supplementation (100 000 U/month), individualization of DCC [19] and oral calcium or oral alfacalcidol therapy; cinacalcet was introduced as a last alternative. PTH lowering therapies were decreased for patients with acute comorbidities and SPD. Surgical 7/8 PTX was performed in patients for whom conservative therapy failed.

The following patient information was recorded: medical history, including cardiovascular events and risk factors; pharmaceutical treatment, including the use of statins, warfarin, vitamin D, cinacalcet, and phosphate binders; and baseline results from standard serum levels laboratory tests. Serum levels were sampled were obtained on a monthly basis for the measurements of: PTH, determined using a second-generation assay (ElecSysG; Roche© Diagnostics, Meylan, France, reference values 10–65 pg/mL), calcium, phosphorus, albumin, and CRP. The rate of bone turnover was evaluated every 6 months using serum levels of bone alkaline phosphatase (b-ALP; chemiluminescence, Beckman© Access, reference values 3.7–20 μg/L), ß-CrossLaps (CTX; Elecsys, Roche© Diagnostics, Meylan, France) and 25-OHD (Abbott© Laboratories. Abbott Park, Illinois, U.S.A.). For laboratory tests, blood samples were obtained in a non-fasting state before a mid-week dialysis session, with all serum levels measured from the same blood draw. Single pool Kt/V was calculated using the second-generation logarithmic formula of Daugirdas. Daily protein intake was measured by calculating normalized protein nitrogen appearance (nPNA). Common laboratory analyses were performed by the Grand Vallon Laboratory (NOVESCIA© Lyon, France).

### Statistical analysis

The data of patients who experienced more than one episode of PTH decrease were excluded from the analysis. The mean ± standard deviation (SD) was calculated for all variables. Baseline characteristics among patients in each group (MPD, PTX, SPD, and NPD) were evaluated using Fisher’s exact test, with a regression correlation applied when necessary.

The 60-month survival was analyzed using Kaplan-Meier and Cox multivariable proportional hazards models. Univariate analysis was used to identify variables associated with all-cause mortality. All identified variables were included in a multivariate Cox analysis, using a backward elimination to identify independent factors associated with mortality, using a *p* <0.05 to retain a variable. The relative risk of mortality was expressed as a hazard ratio (HR).

Data were censored for kidney transplantation, transfer to another dialysis center or lost to follow-up, and at the end of follow-up. All statistical analyses were performed using MedCalc software version 11.5.1.0 (MedCalc Software, Ostend, Belgium). A p value ≤0.05 was considered significant in all analyzes.

## Results

**Fig. 2:**
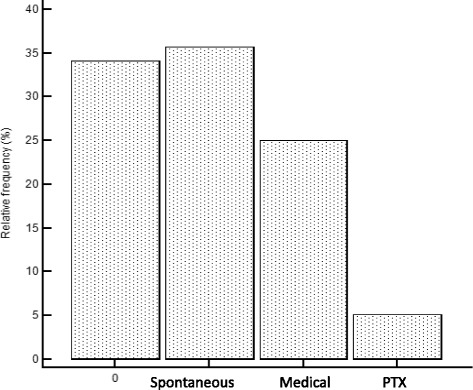
Relative frequency of patients in the 4 groups. 0 = no PTH decrease. Spontaneous = Spontaneous PTH decrease. Medical = medical PTH decrease. PTX = PTX patients

Among the 235 patients receiving HD in January 2010, 21 were excluded for dialysis vintage <6 months and 10 for previous PTX. The data of another seven patients were excluded at the end of the study due to more than one episode of PTH decrease. The distribution of the 197 remaining patients among the four causative groups of a PTH decrease (NPD, SPD, MPD, and PTX) is shown in Fig. 2 and summarized as follows: NPD, 34 % (*n* = 67); SPD, 35 % (*n* = 70); MPD, 25 % (*n* = 50); and PTX, 6 % (*n* = 10). Baseline characteristics of patients among the four groups are reported in Table 1. The characteristics of patients requiring PTX during the study period were quite different from those of patients in the other groups. Namely, patients in the PTX group were younger and had a lower prevalence of cardiovascular disease, diabetes, and malnutrition-inflammation syndrome, but with higher baseline serum PTH levels and DCC, as well as a more frequent use of alfacalcidol and cinacalcet. Compared to patients in the SPD and NPD group, patients in the MPD group had started HD more recently (i.e., shorter dialysis vintage) and had a lower prevalence of cardiovascular disease and diabetes. Specific comparison of baseline characteristics among patients in the SPD and MPD groups is reported in Table 2. Compared to patients in the MPD group, patients in the SPD group showed lower serum PTH and albumin levels, lower DCC and higher CRP levels. The median serum PTH value decrease was −75 % in the SPD group and −70 % in the MPD group.

**Table 1:** Baseline characteristics

	No PTH decrease *N* = 67	Spontaneous PTH decrease *N* = 70	Medical PTH decrease *N* = 50	Surgical PTX *N* = 10
Age (years)	66.8 ± 14	71.2 ± 12	64.1 ± 16	51 ± 15 *
Female gender (%)	46.3	27.1 *	53.1	40
Dialysis vintage (months)	75.1 ± 98	71.5 ± 73	53 ± 61 *	90.5 ± 94
Diabetes (%)	37.3	41.4	24.5 *	0 *
Cardiac disease (%)	28.4	30	10 *	10 *
Peripheral vascular disease (%)	27	25	8 *	0 *
Stroke (%)	9	13	4 *	0 *
Cancer (%)	7	16	16.3	10
BMI (kg/m^2^)	25.8 ± 5	24.7 ± 5	24.6 ± 4	25 ± 4
Body weight (kg)	71.1 ± 18	69.7 ± 16	67.4 ± 13	70.5 ± 17
Dialysis session time (min)	330 ± 95	322 ± 90	327 ± 92	410 ± 100 *
Central catheter (%)	13.4	20	12.5	10
HDF (%)	40.3	40.3	39.6	12.5 *
Dialysate calcium (mmol/L)	1.5 ± 0.16	1.47 ± 0.16	1.53 ± 0.14	1.7 ± 0.18 *
spKt/V	2.3 ± 0.7	2.1 ± 0.6	2.4 ± 0.7	2.7 ± 1 *
nPNA (g/kg/day)	1.42 ± 0.6	1.14 ± 0.2	1.3 ± 0.4	1.43 ± 0.3
PTH (pg/mL)	198.6 ± 152	218 ± 126	280 ± 144	498 ± 315 *
Calcaemia (mmol/L)	2.26 ± 0.14	2.23 ± 0.14	2.25 ± 0.12	2.31 ± 0.12
Phosphataemia (mmol/L)	1.42 ± 0.3	1.34 ± 0.36	1.36 ± 0.3	1.26 ± 0.2
b-ALP (μg/L)	18 ± 10	21 ± 15	19 ± 11	35.6 ± 27 *
25-OHD (nmol/L)	106 ± 49	112 ± 39	104 ± 41	120 ± 28
Serum Albumin (g/L)	33.5 ± 4	31.1 ± 4	33.8 ± 4	36.8 ± 3 *
C-reactive protein (mg/L)	11.7 ± 16	14.6 ± 18	7.6 ± 8	4.3 ± 3 *
Cholecalciferol (%)	90.5	92.3	93.3	90
Alfacalcidol % (μg/week)	10 (2.2)	10 (1.6)	25 (2.5)	50 (2.9) *
CacCO3 % (g/day)	27 (1.2)	16 (1.1)	30 (2)	30 (2.4)
Sevelamer % (g/day)	39 (1.8)	21 (1.2)	37 (1.8)	20 (2)
Cinacalcet % (mg/day)	22 (35)	7 (30)	6 (39)	50 (60) *

*P < 0.05; **P < 0.005 with at least one other value

**Table 2:** Comparison between spontaneous PTH decrease (SPD) and medical PTH decrease (MPD) groups

	SPD		MPD	
	T-1	T-2	T-1	T-2
PTH pg/mL median (95 % CI)	330 (125 – 785)	80 (10 – 200)**	490 (190 – 1230)	147 (122 – 178) **
Calcaemia mmol/L	2.22 ± 0.15	2.2 ± 0.12	2.35 ± 0.18	2.24 ± 0.13 *
Phosphataemia mmol/L	1.38 ± 0.4	1.21 ± 0.4 *	1.45 ± 0.4	1.35 ± 0.4 *
Serum albumin g/L	33 ± 4.5	28.6 ± 4 **	34 ± 4	34.2 ± 4.3
CRP mg/L median (95 % CI)	8.1 (8 – 50)	105 (15 – 300) **	3,6 (0 – 25)	4,5 (0 – 29)
Dialysate calcium mmol/L	1.51 ± 0.17	1.35 ± 0.2 *	1.52 ± 0.18	1.65 ± 0.15 *
Mortality	73 %		24 %	
Median survival time	10 months		24 months	

* *P* < 0.05; ** *P* < 0.005 between T-1 and T-2

After the initial serum PTH value decrease, PTH level remained stable after 6 months in the MPD group, but tended to increase in the surviving SPD patients. This could be due to therapeutic adjustments or acute comorbidity improvement. No significant serum PTH level increase was observed using the same criteria (>50 % from baseline in less than 3 months) in the NPD group.

The overall mortality rate among patients in the SPD group was 73 %, compared to a rate of 24 % among patients in the MPD group (*p* < 0.001), with a median survival time of 10 months for the SPD group and 24 months for the MPD group (*p* < 0.001).

The comorbid conditions and therapeutic changes associated with a decrease in PTH in the SPD and the MPD groups are reported in Tables 3 and 4, respectively. Among patients in the SPD group, the main acute comorbid conditions were peripheral vascular and cardiac complications, sepsis, fractures, and cancers with an increase in serum CRP level (from 14.3 ± 18 to 132 ± 90 mg/L) and a decrease in serum albumin (from 33 ± 4.5 to 28.6 ± 4 g/L). In the MPD group, the main therapeutic change associated with a decrease in PTH was an increase in DCC, either independently or in association with cinacalcet therapy, and less frequently associated with the prescription of oral calcium or alfacalcidol.

**Table 3:** Comorbid conditions associated with spontaneous PTH decrease (SPD)

SPD *N* = 68 (35 %)	
Peripheral vascular disease-arterial by-pass-amputation (*n* = 15)	
Severe sepsis (*n* = 10)	
Factures (*n* = 10)	
Osteoarthritis (*n* = 10)	
Cancer (*n* = 7)	
Cachexia (*n* = 4)	
Gut infection (*n* = 4)	
Cardiac disease (*n* = 3)	
Postoperative (*n* = 3)	
Stroke (*n* = 2)

**Table 4:** Changes in treatments associated with medical PTH decrease (MPD)

MPD *N* = 50 (25.5 %)	
↑ CCD 1.5 to 1.75 mmol/L (*n* = 25)	
↑ CCD from 1.25 to 1.5 mmol/L (*n* = 4)	
↑CCD from 1.5 to 1.75 mmol/L + Cinacalcet (*n* = 10)	
Oral calcium (*n* = 6)	
Alfacalcidol (*n* = 5)	

**Fig. 3:**
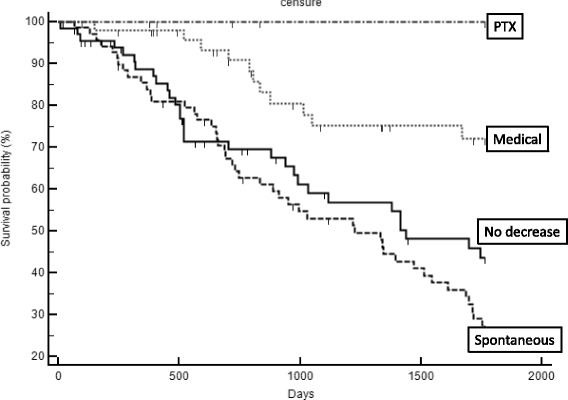
Survival curve (Kaplan-Meier) comparing patients of the 4 groups

**Fig. 4:**
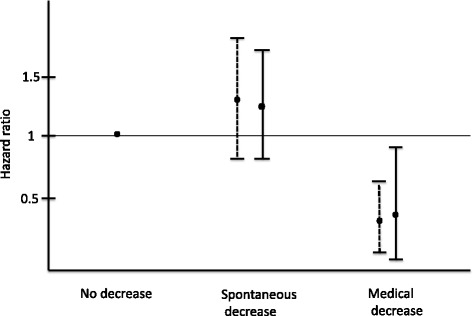
Hazard Ratio of mortality according to the PTH decrease groups. No decrease group as reference. ------ Univariate. ____ Multivariate Cox

The Kaplan-Meier univariate survival analysis is summarized in Fig. 3. Data from the PTX group was not included in the analysis due to the limited number of patients in this group, with no incidence of death over the study period. The rate of mortality was higher among patients in the SPD group than among patients in the MPD group (p < 0.001), with a HR of 3.5 (range, 2–6) for the SPD group and a HR of 1.36 (range, 0.8– 2.3) for the MPD group. Moreover, the mortality rate among patients in the MPD group was lower than the rate among patients in the NPD group (HR, 0.38; range, 0.22–0.66; *p* < 0.001). Using a multivariate Cox model, the mortality rate among patients in the MPD group remained significantly lower than the rate among patients in the NPD group (HR, 0.42; range, 0.2–0.87; *p* = 0.01) or the SPD group (HR, 0.36; range, 0.18–0.73; *p* = 0.004), after adjustment for age, dialysis vintage, peripheral vascular disease, stroke, diabetes, cardiac disease, serum albumin, central venous catheter and warfarin use. The HR of mortality, for both the univariate and multivariate Cox model, are shown in Fig. 4.

## Discussion

To our knowledge, this is the first study to stratify HD patients according to their underlying causes of decreases in serum PTH levels in patients on HD, and the resultant effects of decreases in PTH levels on the survival outcomes of these patients. Among the 197 HD patients forming our study group, a decrease in PTH level was associated to an acute comorbid condition in one-third of patients (SPD group) and to a change in medical therapy (MPD group) or PTX in another one-third of patients. Survival over the 60-month period of the study was lower among patients in the NPD and SPD groups, compared to the survival rate among patients in the MPD and PTX groups. Even after adjustment for possible influencing variables, the prognosis remained dramatically lower among patients in the NPD and SPD groups.

### Regulation of PTH

SHPT is normally observed in patients with CKD stage 4 to 5, resulting from a prolonged decrease in serum levels of calcium and vitamin D (1,25(OH)_2_D_3_) or an increase in serum phosphate level. SHPT in this clinical population has been associated to an increase in PTH gene expression, synthesis, and secretion, and to a proliferation of parathyroid cells in chronic cases [20]. More recently, the role of Klotho deficiency, resistance of parathyroid cells to fibroblast growth factor (FGF)-23 action, due to a decrease of fibroblast growth factor receptor 1 expression [21], and a decrease of serum 1,25(OH)_2_D_3_ due to the excess FGF-23 synthesis, have been postulated to influence SHPT in patients with end-stage CKD [22].

Tertiary hyperparathyroidism, with an increase of the set-point calcium-PTH and frequent hypercalcemia, represents the response of the autonomous function of one or more parathyroid glands to long-standing SHPT [23]. The consequences of prolonged SHPT include bone disease, previously known as osteitis fibrosa, with an increased risk for radiological demineralization [24], fractures [25] and brown tumors [26], as well as an increased risk for cardiovascular calcification [5] and mortality [2].

### High PTH-related morbidity and mortality

In a recent metaanalysis of HD patients, Natoli et al. reported that only a high serum PTH level was associated with mortality [27]. Using the Fresenius US database, Block et al. reported an increased rate of mortality only among patients with a serum PTH level >600 pg/mL in an adjusted model, whereas in an unadjusted model, only low PTH levels were associated with a higher rate of mortality [11]. The same findings were confirmed for patients with CKD not on dialysis by Kovesdy et al. [7] and Nakai et al. in Japan [28]. The mechanism of PTH toxicity is poorly understood. However, an elevated calcium x phosphorus (Ca x P) product associated with tertiary HPT and a high serum PTH level, associated with high serum FGF-23level, have been associated with a risk for progression of cardiovascular calcification [5, 29].

### Low PTH-related mortality

In patients with CKD and in those on HD in particular, both low and high serum PTH levels have been associated with a higher rate of mortality [30]. Twenty years ago, Fournier et al. reported that a persistently low PTH state could result from aluminum intoxication, older age, diabetes, and excessive calcium and vitamin D use, as well as being frequently observed in patients undergoing peritoneal dialysis [31, 32]. The toxicity of a serum level of PTH that is within normal range, and even judged to be too low in HD patients, is probably related to an underlying condition, such as hypo phosphatemia and poor nutrition [33]. Over the last century, the excessive use of calcium and vitamin D to treat hyperphosphatemia and prevent SHPT has led to iatrogenic ABD, with the resulting hypercalcemia increasing the risk of extraosseous calcifications and mortality [8, 34]. Based on the data in the Taiwan HD registry, Lin et al. found that a low serum PTH level increased the risk of mortality usually when associated with hypercalcemia [35]. Today, in industrialized countries, non-calcium phosphate binders and higher serum PTH targets are used to prevent an excessive calcium load and hypercalcemia and, therefore, iatrogenic ABD is infrequently reported [4].

In addition to iatrogenic causes of low serum PTH, Fukagawa et al. suggested that a low serum PTH level may reflect a malnutrition status, which would explain the poor prognosis of these patients [36]. In HD patients, Mehrotra et al. reported an inverse relationship between age and serum levels of PTH [37], with a further association between low serum albumin levels and low PTH level in a Japanese cohort of HD patients [38]. The malnutrition-inflammation syndrome was associated with low PTH levels and poor outcomes [10, 39]. In their study of 748 HD patients, Dukkipati et al. also reported that low PTH (<150 mg/mL) was associated with protein energy wasting and inflammation. They further reported that a PTH level between 100 and 150 pg/mL to be associated with a greater survival compared to other PTH levels [40].

The effect of inflammation of PTH levels was described by Bologa et al. who reported that, in vitro, PTH secretion is suppressed by IL-6 [41], with IL-1ß inhibiting PTH secretion in parathyroid tissue slices [42], with IL-1 specific receptors upregulating the calcium-sensing receptor mRNA. Therefore, the KDIGO experts recommend maintaining serum PTH levels in a large range, from to 2–9 times the upper limit of the assay in HD patients to lower the risk of severe osteitis fibrosa and ABD [2] and to favor survival, based on the U-shape survival curve associated with serum PTH level. Hence, it is advisable to treat SHPT in HD patients in order to prevent health complications related to SHPT and improve survival, but without surpassing target levels due to the risk of ABD and related hypercalcemia.

### Treatment for SHPT

Available treatments for SHPT include the use of native vitamin D [43], oral calcium [44], high DCC [19], calcitriol and analogs [45], and cinacalcet [46], as well as surgical PTX [47]. In a randomized controlled study evaluating the relative effectiveness of using calcium salt and non-calcium phosphate binders in the treatment of SHPT, outcomes were frequently worse for patients receiving calcium salts due to an inadequate calcium load, resulting in higher calcemia and more frequent episodes of hypercalcemia and extraosseous calcification [48]. By comparison, observational studies have described the use of oral calcium to be associated with better outcomes when prescribed adequately [49, 50]. The outcomes associated with the use of DCC are difficult to evaluate as few centers adjust DDC to levels of calcemia and bone turnover markers in patient-specific ways as we do in our center [19]. However, an observational study in a French regional cohort of HD patients identified no deleterious effects of high DCC (1.75 mmol/L) when prescribed for patients with hypo- or normo-calcemia [51].

A large observational study based in the United States (US) identified the use of calcitriol, and of its analogs, as being effective in lowering the risk of mortality in HD patients [52], as we previously reported in our regional cohort [53]. Randomized trials did not provide evidence of a survival advantage with the use of cinacalcet therapies, compared to high doses of calcitriol, or of its analogs [54]. In secondary analyzes of the data from this trial, cinacalcet therapies were associated with a lower incidence of fractures [55] and calciphylaxis [56]. The long-term prognosis of cinacalcet therapies, compared to PTX, has yet to be evaluated. In our study, we reported better outcomes among patients in the MPD group than among those in the NPD and SPD groups who were using calcium therapy (DCC and oral calcium), cinacalcet and alfacalcidol. When calcium, vitamin D or cinacalcet are used to lower serum levels of PTH, it is not possible to differentiate the impact of these therapies on survival from the impact of changes in serum levels of vitamin D or the Ca x P product on survival.

Low PTH levels after surgical PTX has been reported to improve outcomes in a US-based study of HD patients [57]. However, Konstantidinis et al. recently reported that only a few patients meeting the biological criteria for PTX actually accepted the surgery in the US, even if the PTX allowed achieving biological KDOQI targets of PTH [58]. In Japan, the survival rate after PTX has been reported to be very good, with an early mortality rate of only 0.15 % [59]. In our study, patients who underwent surgical PTX had excellent mid-term outcomes; however, the majority of these patients underwent kidney transplantation by the end of the study period and, therefore, the longer term survival could not be evaluated.

### Causes and impact of a decreased PTH level

In a French observational study, using the KDIGO biological target of PTH level, Merle et al. reported that a decrease in serum PTH level after one year, including both changes from normal to low levels and from high-to-low levels, was associated with a higher rate of cardiovascular-related mortality [16]. Among factors associated with a higher risk of mortality, Merle et al. identified a high serum CRP level and a high DCC (1.75 mmol/L). Of importance is their finding that mortality risk increased when high DCC and a low albumin level were associated with a PTH decrease; high DDC was not associated with an increased rate of mortality among patients in whom PTH level remained in the low range. However, Merle et al.’s study was based only on biannual PTH sampling, and information on acute comorbid conditions or changes in therapeutic intervention was not available between sampling periods. Moreover, high DCC was prescribed in their study based on usual care rather than being individualized based on PTH values. We consider that using the KDIGO target level (i.e., low-normal-high level classification) to study PTH variations may be questionable as a small variation in PTH level could be associated with a change in target zone classification. In the COSMOS study of 4500 European HD patients evaluated at 6-month intervals, Fernandez-Martin et al. reported that a serum PTH decrease was not associated with an increased rate of mortality when baseline PTH levels were within the KDIGO range of normal (168 – 674 pg/mL), with the risk of mortality significantly increasing among patient with a baseline PTH level <168 pg/mL [15]. Again, no data on acute comorbid conditions or change in therapeutic intervention was provided. We hypothesize that a decrease in PTH among patients in the low level KDIGO range would reflect an acute comorbid condition, rather than a medical decision on treatment, which would explain the finding of a higher rate of mortality. In their large US-based cohort study of HD patients, Streja et al. reported that an increased in serum PTH level from <150 pg/mL to 150–300 pg/mL was associated with a lower risk of mortality [60]. Once again, this was an observational study with no information on the underlying cause of variation in PTH level provided.

### Limitations

The fact that we conducted a monocentric study with a small number of patients limits the generalization of our results. However, the treatment using a strategy that has been homogenous for 20 years, as well as using monthly serum PTH sampling, allowed us to identify factors associated with variation in serum PTH levels.

## Conclusion

In our study, we determined the survival outcomes associated with different causes of a decrease in PTH level in HD patients. A SPD, resulting from an acute comorbid condition, is associated with poor survival outcomes, with MPD and PTX being associated with lower rates of mortality. The poor outcomes associated with SPD should not lead to underestimate the risk of SHPT that is highly prevalent in HD patients. Medical or surgical therapy should be implemented in order to prevent a negative impact of SHPT on bone and vascular health.

## Abbreviations

ABD: Adynamic bone disease
BMI: Body mass index
CKD: Chronic kidney disease
CRP: C - reactive protein
CTX: Beta-CrossLaps
DCC: Dialysate calcium concentration
DOPPS: Dialysis outcomes and practice patterns study
HD: Haemodialysis
HDF: Haemodiafiltration
MPD: Medical parathyroid hormone decrease
NPD: No parathyroid hormone decrease
nPNA: Normalized protein nitrogen appearance
PTH: Parathyroid hormone
PTX: Parathyroidectomy
SD: Standard deviation
SHPT: Secondary hyperparathyroidism
SPD: Spontaneous parathyroid hormone decrease
